# Diversity and Distribution of Braconidae, a Family of Parasitoid Wasps in the Central European Peatbogs of South Bohemia, Czech Republic

**DOI:** 10.1673/031.010.1601

**Published:** 2010-03-13

**Authors:** Aurel I. Lozan, Sergey Belokobylskij, Cees van Van Achterberg, Michael T. Monaghan

**Affiliations:** ^1^ Biology Centre, Institute of Entomology, Academy of Sciences of the Czech Republic, Branišovská 31, CZ-370 05 České Budějovice, Czech Republic; ^2^ Zoological Institute, Russian Academy of Sciences, Universitetskaya nab., I St. Petersburg 199034, Russia; and Museum and Institute of Zoology, Polish Academy of Sciences, Wilcza 64, Warszawa 00-679, Poland; ^3^ Department of Entomology, Nationaal Natuurhistorisch Museum (Naturalis), 2300 RA Leiden, Netherlands; ^4^ Leibniz-lnstitute of Freshwater Ecology and Inland Fisheries (IGB), Müggelseedamm 301, 12587 Berlin, Germany

**Keywords:** Central Europe, Hymenoptera, Braconidae, ecology, faunistics, tyrpho-classification

## Abstract

An ecological overview of seven years investigation of Braconidae, a family of parasitoid wasps (Hymenoptera: Ichneumonoidea) and a tyrpho-classification of parasitoids in peatbog areas of South Bohemia, Czech Republic are given. A total of 350 species were recorded in investigated sites, but only five tyrphobionts (1.4%) are proposed: *Microchelonus basalis, Microchelonus koponeni, Coloneura ate, Coloneura danica* and *Myiocephalus niger.* All of these species have a boreal-alpine distribution that, in Central Europe, is associated only with peatbogs. Tyrphophilous behaviour is seen in at least four (1.1%) species: *Microchelonus pedator, Microchelonus subpedator, Microchelonus karadagi* and *Microchelonus gravenhorstii;* however, a number of other braconids prefer peatbogs because they were more frequently encountered within, rather than outside, the bog habitat. The rest of the braconids (342 species, 97.5%) are tyrphoneutrals, many of them being eurytopic components of various habitats throughout their current ranges. Lists of tyrphobiontic braconids and a brief commentary on species composition, distributional picture of actual ranges, and parasitoid association to bog landscape are provided. Being true refugial habitats for populations in an ever-changing world, peatbogs play a significant role in harboring insect communities.

## Introduction

South Bohemian peatbogs are isolated, paleorefugial habitats with unique flora and fauna characteristic of oligotrophic mires. These habitats have developed under specific conditions, predominantly within a “forest tundra climate” (Spitzer 1994). Parasitic braconid wasps (Hymenoptera: Braconidae) are among the most often encountered components of these ecosystems, and their diversity in peatlands implies the presence of complex interactions among plants, hosts, and parasitoids. Despite previous work (e.g. Enderlein 1908; Krogerus 1960; Finnamore 1994; Papp 1982; Tereshkin 1996; Lozan and Tobias 2002), faunistic and taxonomic analyses of peatbog braconids remain limited. Little is known about host-parasitoid linkages in peatbogs (Krogerus 1960; Chalmers-Hunt 1969; Havel 1970; Bourn and Warren 1997), despite their potential importance to bog ecosystems and implications for bogland conservation.

Typically, insects inhabiting peatbogs are classed ecologically as tyrphobionts, tyrphophiles, or tyrphoneutrals (e.g. Spitzer 1994; Spitzer et al. 1999; Spitzer and Danks 2006), the former being of conservation interest because of their strict dependence on the bog environment. To date, there is no existing ecological classification of Braconidae inhabiting peatbogs. A critical problem is that taxonomic descriptions and records from Central Europe (and elsewhere) do not always include information about habitat, and the records may be false as misidentifications and misinterpretations are rather common in the many groups that are not properly revised. Additionally, limited, and sometimes controversial, data on species ranges and habitat preferences often hinder our understanding of species' bog affinity (for members of the genus *Microchelonus* as an example, see Telenga 1941; Shenefelt 1973).

Here we sampled the braconid parasitoid fauna from 7 sites in South Bohemia and used curated specimens from 7 museums in order to compile a list of tyrphobiontic species (full list of species provided in the Appendix) based on all available geographical information, field data, museum material (Palaearctic only), and direct correspondence with specialists, collection curators and collectors. This is the first large-scale survey of braconid parasitoids in Central Europe, with particular emphasis on the bogs of South Bohemia in the Czech Republic.

## Materials and Methods

### Site descriptions

**Figure 1:**
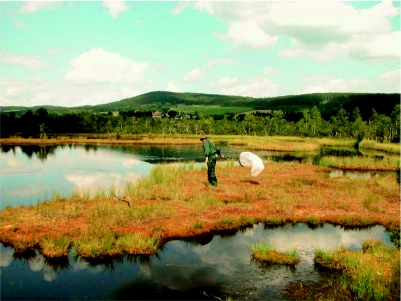
The central parts with a lake of the Chalupská Slat' bog (900 m) in the Šumava Mts. High quality figures are available online.

**Figure 2:**
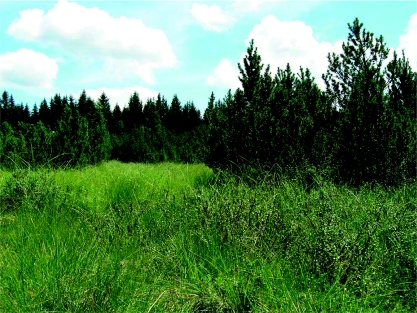
The Jezerní Slat' bog (1075 m) in the Šumava Mts.: *Betula nana* and *Pinus mugo* s. lat. visible in the front view. Higher quality figures are available online.

The 7 examined bogs span an altitudinal gradient from lowland (470 m) to mountain raised peatbogs (1120 m) and are dominated by *Sphagnum* mosses, ericaceous *Vaccinium* shrubs, and, to some degree, forest trees, particularly mountain and bog pine (complex of *Pinus mugo* s.l.) (Spitzer 1994; Spitzer and Jaros 1993, 1998; Spitzer et al. 2003; Bezděk et al. 2006; Kučera 1995). Some of the investigated bogs of South Bohemia, particularly in the Treboň Basin, were partially eroded by former human activities that left a succession of vegetation in clearings and hollows after peat exploitation. Following is a brief description of the bogs used in the study (see Figures 1–2): (1) Červené Blato (472 m, 331 ha), in the Třeboň Basin near Š almanovice, is a transient peatbog forested by bog pine (*Pinus rotundata*) and shrubs of *Vaccinium* spp. and *Ledum palustre;* (2) Mrtvý Luh (740 m, 310 ha) near Volary is a core zone of Š umava National Park. It is a valley peatbog, surrounded by forest and relatively isolated by mountains. *Sphagnum* spp., *Vaccinium uliginosum,* and *Eriophorum vaginatum* comprise the unforested parts of the bog, with some areas of dwarf forest of *P. mugo* s. lat; (3) Velká Niva bog (750 m, 120 ha) is near Lenora in the Sumava Mountains. In outer areas, it is a waterlogged spruce forest, and the central area is open forest of *P. mugo* s. lat. with patches of *Betula pubescens;* (4) Chalupská Slat' bog (900 m, 116 ha) near Borová Lada is also a core zone of Šumava National Park. It is an intermediate between valley bog and mountain raised bog, with a central lake, with *Betula* spp. and *Carex* spp. in the bog margins, and with an outer ring of mountain pine forest; (5) Jezerní Slat' is a montane upland peatbog (1050 – 1075m, 190 ha), a core zone in the Š umava National Park, covered with large islands of *Pinus mugo* s. lat. and treeless areas of shrubs of *Betula nana* and *Vaccinium* spp. in cotton-grass layers; (6) Rokytská Slat' (1073 – 1119 m, over 250 ha), a core zone in the Sumava Mts., is a typical mountain-type of raised peatbog, where small, central bog-lakes are surrounded by mountain pines (*P. mugo* s. lat.), with spruce trees and dwarf birches of *B. nana* and *Vaccinum* spp. in opened areas; (7) Luzenská (= Hranični) Slat' (1130 – 1120m), a core zone in the Sumava National Park, is a complex of several small, raised peatbogs, with several small bog-lakes and shrubs of *Vaccinium* spp. and *P. mugo* s. lat., surrounded by norway spruce forest (*Picea excelsa*). All but Luzenská (= Hranični) Slat' are National Nature Reserves.

Data are included for three additional peatbog areas in Central Europe using the following museum collections: the National Nature Reserves of Velke Dařko in the Czech-Moravian highlands (material of MMB); the Skřítek peatbog (*ca.* 166 ha) in the Jeseníky Mountains of Silesia (material of MMB); the Pürgschachenmoor bog in Styria, Austria, which is an Naturschutzgebiet and the World Wildlife Fund Nature Reserve and one of the southernmost outposts of boreal peatland ecosystems in Central Europe (see Spitzer et al. 1996) (material of MNHV).

## Parasitoid material

All newly collected specimens were obtained as part of a project studying the diversity and ecology of insects in Central European bogs from 2001–2007. In the field, braconids were collected with a sweep net (d = 40 cm), using 50 net strokes each separated by 1 m along a transect, or occasionally with haphazard sweeps. Samples were taken within bogs and in nearby meadows and forests. Light trapping was conducted in three of the peatbogs using a BL-Pennsylvania black light (8 W), serviced once a week over the growing season (March to November). At Mrtvý Luh, two light traps were used during 2000–2002, and one light trap ran at Velká Niva and Jezerní Slat' from 2005–2007.

Some braconids were obtained from leafspinning Lepidoptera on *V. uliginosum,* although taxonomy of the lepidopteran hosts was undetermined and remained as Tortricidae + Gelechiidae. There were hundreds of cocoon groups (from 15 to 50 white cocoons in a group, usually on *Carex* or *Vaccinium*), mainly of the microgastrine endoparasitoid *Cotesia tibialis* (Curtis), distributed throughout treeless areas of the Mrtvý Luh and Chalupská Slat' bogs. They most likely originated from a rather common lepidopteran host or several hosts, but the precise relationships with the potential host(s) remain unclear. The cocoons of another microgastrine species, *Cotesia gastropachae* (Bouché), were found alongside the remnants of larvae of the lasiocampid moth, *Macrothylacia rubi* (L.), in Mrtvý Luh bog. The parasitoid guilds of bog hosts (Lepidoptera) have been investigated and results will be published elsewhere.

Approximately 7,000 specimens of Ichneumonoidea (Braconidae + Ichneumonidae) were mounted, and most of the material is in the collection of the Biology Centre, Č eské Budějovice, Czech Republic. Certain groups and species were deposited to various museums institutions (ZIM, BMNH, RMNH, MIZW, MMB).

### Habitat comparative study

The degree of association to the bog habitat (i.e. status of tyrphobiont, tyrphophilous or tyrphoneutral) was evaluated by combining distributional records, available habitat data, and newly collected data from peatbogs of South Bohemia. Diagrams of soils derived from former or existing peatbogs in Europe, particulary Great Britain and Ireland (Taylor 1983), were compared in order to understand historical patterns of distribution and follow presumed changes in species ranges. Old series of Braconidae from museum collections (1904–1938, BMNH; 1938–1954, MMB) were analyzed, and checklists (Čapek and Lukáš 1981, 1989; Koponen and Vikberg 1984; Koponen 1984, 1989, 1992; Čapek 1995; O'Connor et al. 1999; Belokobylskij et al. 2003; Belokobylskij 2004; Papp 2005), catalogues (Telenga 1941; Thomson 1895; Fahringer 1930; Shenefelt 1973; Čapek and Hofmann 1996) and keys to species (Fischer 1972, 1976, 1993; Papp 1977, 1995, 2002, 2004; Tobias 1976; Tobias et al. 1986; Van Achterberg 1988; Tobias and Lozan 1995; Belokobylskij and Tobias 1998, 2000) were all used in order to incorporate all available information on species range and habitat affiliation. Other works related to Braconidae of montane moors and/or highlands in Europe were considered as these areas may also harbor endemics or species with certain microclimatic preferences (Hackman and Meinander 1974; Papp 1982; Papp and Rezbanyai-Reser 1999; Zeman and Vaněk 1999; Van Achterberg and Rezbanyai-Reser 2001; Tomanović et al. 2007).

Many species from the samples were compared with the available material (including type material and series from elsewhere) from other collections and museums to exclude taxonomical uncertainties, particularly in difficult taxa. Taxonomically unclear/difficult specimens (some *Aspilota* Förster and *Dinotrema* Förster species) or morphospecies were not included in our analysis. Series of both males and females of some species have been analysed separately to avoid the problem of linking sexes in sexually dimorphic species.

## Results and Discussion

### Diversity and association to peatbogs

A total of 350 species from 19 subfamilies and 76 genera were recorded in the samples from peatbogs of South Bohemia during 2001–2007 (see the Appendix for a complete list). Most of the Braconidae, 222 species (64%), are new records in the Czech Republic, although almost all species are known from neighbouring countries (Čapek and Lukáš 1989; Čapek and Hofmann 1997; Van Achterberg 2002; Belokobylskij et al. 2003; Belokobylskij 2004; Papp 2005). The most numerous taxa belong to the subfamilies Alysiinae (94 new country records) and Microgastrinae (44 new country records), comprising approximately 63% of all new faunistic records for the country.

The vast majority of species were also found in adjacent or “non-bog” areas and as a result, only 5 species (1.4%) are proposed here as tyrphobiont taxa (Table 1): *Microchelonus basalis* (Curtis), *M. koponeni* Tobias, *Coloneura ate* (Nixon), *C. danica* Griffiths and *Myiocephalus niger* Fischer. These species were never present outside of bog habitat in Central Europe and thus appear to be obligatory components of peatlands. *M. niger* occurs in northern Europe and Asia but the remaining four species are recorded only from Europe. They are typically boreal and arctic/cold-adapted species, and here display a clear, narrow association with bogs in Central Europe, as well as a probable dependence on edaphic conditions of the bog habitat.

Four additional species (1.1% of the total), *Microchelonus pedator* (Dahlbom), *M. subpedator* Tobias, *M. karadagi* Tobias and *M. gravenhorstii* (Nees) are here classified as tyrphophiles, being frequently encountered within peatbogs and only rarely in wet meadows or forests nearby. The remaining 341 species (97.5%) are considered tyrphoneutrals, being more or less widely distributed and not only found in peatlands. Most of them are eurytopic or opportunistic species, often abundant and locally dominant.

**Table 1:**
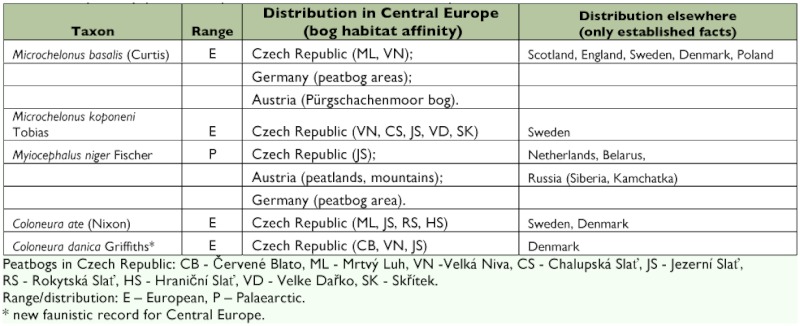
Proposed tyrphobiontic species of Braconidae in Central Europe

### Taxonomic composition of samples

In peatbogs, the cyclostome complex of subfamilies is represented by the Braconinae with three genera and 23 species, the Rogadinae by 15 species of only one genus (*Aleiodes* Wesmael), the Exothecinae s. 1. by five genera and ten species, the Doryctinae by three species, and the Rhyssalinae by two species. Many of these are associated with forest areas in boreal or temperate zones, being widely distributed in Palaearctic and/or Holarctic realms. The polymorphic Euphorinae, present in all studied bogs with 16 genera and 40 species, were locally abundant and also found in other wetlands throughout the country. The carapace-bearing Cheloninae wasps were present in peatbogs with four genera and 20 species (Lozan 2006; Lozan et al. unpublished data). Seemingly, most members of the genus *Microchelonus* Szépligeti are obviously confined to bog habitat (Lozan and Tobias 2002, 2006), however, their real diversity and ecological affinity to peatbogs are still poorly understood.

The Microgastrinae is the second speciose subfamily, with 8 genera, 63 species and containing koinobiont endoparasitoids of Lepidoptera. Several of the species were the most abundant braconids among all samples. Remarkably, the species *Cotesia tibialis* (with a trans-Palaearctic distribution) presented almost everywhere and was extremely abundant in Mrtvý Luh and Chalupská Slat'.

There were 142 species (40% of all collected braconids) within the clade of subfamilies Opiinae + Alysiinae, koinobiont endoparasites of cyclorrhaphous Diptera. Opiinae were represented by 6 genera and 26 species, while 24 genera and 124 species of Alysiinae were found in investigated peatbogs. Most of these are newly recorded here for the fauna of Czech Republic. Such genera as *Alysia* Latreille, *Idiasta* Förster, *Anisocyrta* Förster, and *Phaenocarpa* Förster are known to occur in the northern part of the northern hemisphere (most taxa are boreal with Holarctic range) (Wharton 1986) and were present in almost all of the investigated bogs.

### Nocturnality

Crepuscular and/or nocturnal flight activity is well-known in several subfamilies of braconids (Gauld and Huddleston 1976; Jakimavičius 1979; Čapek and Lukaš 1981; Huddleston and Gauld 1988; Quicke 1992; Lozan 2002, 2004). From 26 species in our light trap samples, only one tyrphobiont (*M. niger*) was present, while the rest seem to be tyrphoneutrals, being widely distributed and not restricted to the bog habitat (Lozan 2002). Only two species are egg-larval idiobionts (*Diospilus* Haliday) of coleopteran larvae (Belokobylskij 1996), and the rest of the species are endoparasitoid koinobionts of either Lepidoptera or Coleoptera, or, rarely, Diptera.

### Tyrpho-classification of Braconidae

There is a general lack of ecological data, particularly habitat affinity, for most Hymenoptera Parasitica; therefore, the tyrpho-classification is an attempt to distinguish taxa with different degrees of association to peatbogs. In the samples there were many species, somewhat locally abundant, overwhelmingly dominant in peatbogs and rarely (if at all) present in other habitats within the country; however, recorded habitat data from elsewhere range from forests to meadows and various wetlands (including marshes and peatbogs).

Quantitative indexes in bog samples have not always been reflecting the true connection to peatbogs, as many eurytopic species were numerous within rather than outside and as other species have only been encountered in investigated sites. Several Holarctic species (*Ontsira imperator* Haliday, *Ichneutes reunitor* Nees, *Anisocyrta perdita* Haliday), ranging around the circumboreal forests in the northern hemisphere have also been recorded from peatbogs and, likewise, the other forest type species in the Palaearctic region (*Macrocentrus resinellae* (L.), *Bracon hylobii* Ratzeburg, *Coeloides abdominalis* (Zetterstedt) etc.), which are widely distributed and not connected with peatbogs, could easily be mistreated as highly associated to bogs. Usually, these species are cold-adapted and rather abundant in northern areas (Van Achterberg 1986; Koponen 2000; Hance et al. 2007), so their habitat pictures include not only forests, but also wetlands (swamps, marches, peatbogs), boggy forested areas and upland (alpine) meadows southward, being ecologically confined to their potential forest hosts. Such boreo-montane elements as *Alysia fuscipennis* Haliday, which is a European species occurring in uplands and montane meadows (sometimes abundant), has also been found in peatbogs; however, most specimens were collected in the wet meadows nearby. The Holarctic euphorine species *Myiocephalus boops* Wesmael is recorded from the *Pinetum-sphagnosum* community (collected by Malaise trap, abundant) in peatlands of Belarus, swept also from *B. nana* in boggy areas of Finland (Koponen and Vikberg 1984) and in taiga-forest of Russia (Buryatia, Yakutia, Kamchatka) (Belokobylskij and Tobias 2000). Nevertheless, all these species are not typical for peatlands, so they are incuded in the tyrphoneutral category.

### Tyrphobiontic Braconidae: Species breakdown and habitat affinity

This is a very specific and characteristic ecological group restricted to bogs, including five braconid species in our samples, with no evidence of occurrence in other habitats in Central Europe (*M. niger* is recorded also from high mountain, however, boggy areas). All these species seem to require microclimatic conditions typical for peatbogs. There is no obvious evidence these species are associated closely with bogs through their hosts (and it should not be obligatory), but edaphic conditions are probably among basic ecological determinants in their successful development. All these species are cold adapted with affinities to boreal and subarctic areas in Europe and Asia (Spitzer and Danks 2006). When matching data of localities and/or regions where species were collected, it was discovered that all those areas were either boggy forests (partly open lands with lakes and peatbogs, in Sealand and Jutland [Denmark], for instance, peatbogs within sandy dunes) or areas with severe climate (mountains, boreal forest, tundra). Despite some taxonomic, geographical and ecological uncertainties while working out various series elsewhere, their tyrphobiontic connection is obvious. Many biotopic data used in this classification are from either field observation or from information provided by other collectors. Thus, the species' actual distributional and habitat characteristics within their virtual ranges, including Central Europe, are as follows (also see Table 1):

1) *Microchelonus basalis.* European species (north Europe according to Fahringer 1930): England (southwest, hilly open moorland with blanket bogs of the Exmoor National Park), Scotland (Moulin Moor peatbogs), Finland (northern Lapland, highlands: bogs, forests and lakes), Sweden (bogs of Tyresta National Park, plains of southermost Skĺne province, Degaberga and Hoor [with no details]), Denmark (East Jutland: in dry sandy areas with peatbogs), Germany (in Kettner 1965, but no ecological data; however, the geographical locality corresponds with peatbog areas in northwest Germany, somewhat similar to habitat data from Denmark), Poland (peatbogs with sandy dunes in northeastern parts of the country, somewhat similar habitats to Denmark and Germany; see Enderlein 1908), Austria (a boreal valley peatbog, 632 m, closed by Alps in Styria; see Spitzer et al. 1996), Czech Republic (only from three peatbogs). Host unknown.

Shenefelt (1973), based on Telenga's data (1941), indicates Palaearctic distribution for *M. basalis:* England, Sweden, Russia, Kazakhstan, Iran, Hungary, Finland and Germany. The material is missing and we consider it just a misidentification. Analysis of long series of *M. basalis* in the collection of BMNH (collected in England and Sweden) showed there were several species in fact (*M. basalis, M. pusillus* (Szépligeti) and *M. atripes* (Thomson). We are not sure about the geographical data of labels of some material, so these specimens are excluded (collected in 1931–1935, Marshal coll.' specimen dates 1904). There are two additional specimens from Germany in the collection of BMNH with only label numbers and a locality of a rather common name without any details. Another old specimen in the collection of ZIN is labeled only as “Germany”. The species is included in the checklist of the Braconidae of Germany (Belokobylskij et al. 2003) with reference to Shenefelt (1973), which reffers to Telenga (1941), but the material is missing. No specimens from Russia and Asia were found at all, but it does not exclude the species might occur in boggy areas there.

2) *Microchelonus koponeni.* European species. Described from southeast Sweden in forest and bog areas (Tobias 1995), it has recently been discovered in several peatbogs of Czech Republic (Lozan and Tobias 2002). Long series of this species also have been found in MMB collection from other peatbogs throughout the country in the Czech-Moravian highlands and east-northern Moravia (Silesia, Jeseniky Mts.) (old collection [Cheloninae], 1944–1947, leg. Hoffer, unpublished data). Newer data also come from Sweden (Tyresta National Park, Ungfars mosse bog - materials of the Swedish Malaise Trap Project). Host unknown.

3) *Myiocephalus niger.* Trans-Palaearctic distribution: Austria (boggy areas in mountains, 1000–1150m in Alps), Netherlands (sandy area with lake [“fen”] surrounded by forest), Byelorussia (boggy forests), Russia (northwestern Murmansk region; and Siberia - northern taiga, Kamchatka - boggy forests with lakes). In Czech Republic, only from an upland peatbog (1075 m). Host: presumably ants; however, no direct evidence (Belokobylskij 2000).

4) *Coloneura ate.* European species. It was described from Sweden, the southernmost Skåne province (Nixon 1943) with a maritime climate where the habitat picture relates to large forests, mountains and bogs/peatbogs within. In eastern Denmark (Sealand) its habitat covers boggy areas, similar to that of south Sweden (in Griffiths 1968). In Czech Republic only from peatbogs. It is also mentioned in the checklist of Braconidae of Hungary (Papp 2005), but the species occurrence has not been confirmed. Known host: puparia of *Liriomyza* Mik or *Metopomyza* Enderlein species (Diptera: Agromyzidae) (Griffiths 1968).

5) *Coloneura danica.* European distribution (similar habitat requirements as previous species): Denmark (Sealand, in boggy areas), Czech Republic (only in peatbogs). Known host: *Metopomyza nigrohumeralis* Hendel (Diptera: Agromyzidae), miners on *Carex* (Griffiths 1968).

These five species are highly stenotopic taxa: typically boreal-alpine species, well confined to peatbogs in southern temperate zones and covering various areas northward, and for *M. niger* somewhat extended southward in Siberian or Far Eastern north-south mountain chains or forests and peatbogs.

### Tyrphophilous behaviour

While tyrphobiont braconids are ecologically well characterized by the conditions of the bog habitat, the tyrphophiles are not typical for peatbogs and, therefore, not an easily distinguishable category. Members of the subgenus *Stylochelonus* Hellén species group of the genus *Microchelonus* were quite abundant in peatlands, but found were also in wet meadows nearby. Comparatively widely distributed in central-northern Europe, *Microchelonus* (*S.*) *pedator,* that is considered a rare species, has been collected in abundance in several peat bogs and surrounding meadows (collection of RMNH, Leiden, Netherlands). Hellén (1958) reported *Aphelia paleana* (Hübner) (Lepidoptera: Tortricidae) as host for this species; however, there is no reliable evidence of parasitism (Papp 1997). Taxonomically close to previous species, but lesser known in Europe, is *M.* (*S.*) *subpedator,* which is defined ecologically by the same habitat requirements. *M.* (*S.*) *karadagi,* described from mountain forest-steppe (wood, ‘grass + *Stipa’,* in Tobias 1995) area of Crimea peninsula (Kara-Dag) in Ukraine, was recorded from several peatbogs of Czech Republic (but interestingly never outside the bog habitat at all, see Lozan and Tobias 2002) and from bogs of Tyresta National Park in Sweden (materials of Swedish Malaise Trap Project). Another chelonine species, *Microchelonus* (*Parachelonus*) *gravenhorstii,* is likely to be tyrphophilous, occurring in boggy forests and other peatlands. Long series of this species have been in the collections of RMNH (collected in Netherlands and Spain [peatlands of Galicia]) and MIZW (from peatbogs and boggy forests).

Of course, the list can be extended, and further field data are needed to properly evaluate species belonging to certain categories (see Spitzer and Danks 2006). This is another study case, as tyrphophilous categorization would require long-term and detailed statistical analysis. Not excluded, some mentioned tyrphophiles are in fact tyrphobionts in Central Europe and further complex investigations may change their status.

### Tyrphoneutrals

The rest of the studied Braconidae are considered tyrphoneutrals, most of them having a more general distribution (e.g. generalists, see Lozan 2002; Spitzer and Danks 2006) and/or eurytopic components of various habitats. In peatlands they could be either abundant (especially some *Cotesia* Cameron, *Apanteles* Förster, *Microgaster* Latreille, *Dacnusa* Haliday etc.) or rare species, but never characteristic to bogs. For many of them the bog habitat may be a true refuge, where they can survive and/or find an alternative habitat as a result of changes in environment. However, a certain degree of tyrphobiontic and/or tyrphophile tendency can be found in various groups among Braconidae. As there exists lots of taxonomic issues over species validity and many parasitoid species awaiting discovery, understanding the “shifts” in ecological preference of parasitoids and the mechanisms driving it, e.g. generalist/specialist versus habitat/host affinity, are sometimes very problematic.

### Parasitoids and Bog Landscape

It is already beyond question that the bog environment is rather heterogeneous and provides important habitat conditions for insects (Spitzer and Danks 2006). Presence of patches of trees/shrubs in an opened, treeless area is just emphasizing the role of bog habitat in enhancing parasitoid diversity and raising affinity to the habitat (see Roland 2000). Being mostly niche specific, parasitoids are searching for particular microhabitat or host food niche (see Hawkins 1994), which can be found in a host-rich environment such as peatbogs. Hawkins (1994) also pointed out that hymenopterous generalist (idiobiont) and specialist (koinobiont) parasitoids respond differentially to plant architecture, and plant effects are strongest in natural habitats. Despite the fact that parasitoids may actually respond differentially to vegetation assemblages, the results show that the bog habitat adequately supports this high clade of species richness, where koinobiont braconids are predominant.

## Conclusions

A total of 350 species of Braconidae that were properly identified is only a part of the entomofauna of Hymenoptera Parasitica of Central European peatbogs. While most of the collected braconid parasitoids are tyrphoneutral taxa (341 species, 97.5%), with many of them giving obvious preference to peatbogs, a fewer number of them are associated to bogs, e.g. five tryphobiontic (1.4%) and at least four tyrphophilous (1.1%) species, indicating intrinsic processes characteristic to this habitat only.

As the environment changes, by human intervention or by natural influences, the issues of biodiversity and habitat conservation are extremely important. The bog fauna of braconid parasitoids turned out to be very rich, emphasizing the fact that these habitats provide shelter to numerous guilds and groupings, some of which are unique and many of which remain unknown. No doubt some communities contain many hidden cryptic species, and their real diversity should be much higher, especially among traditionally difficult taxonomic groups such as the parasitic Hymenoptera and their hosts (Quicke 2002).

These fragmented ancient patches of peatbogs harbour highly stenotopic taxa (including “geographical races” among some lepidopterans, see Šula and Spitzer 2000), which together with their parasitoid complexes are of great scientific and conservational values. They represent a patrimonial uniqueness and a clade of tritrophic interrelationships, being true refugiai islands for biodiversity.

## Abbreviations

BMNH: Natural History Museum, London;
RMNH: National Museum of Natural History (Naturalis), Leiden, Netherlands;
ZIN: Zoological Institute, Saint Petersburg, Russia;
MNHV: Museum of Natural History (Naturhistorisches Museum), Vienna, Austria;
MMB: Moravian Museum in Brno, Czech Republic;
MIZW: Museum and Institute of Zoology, Polish Academy of Sciences, Warsaw, Poland

## Appendix 1

Complete list of Braconidae collected in studied peatbogs during 2001–2007.

**Rhyssalinae:**

*Dolopsidea indagator* (Haliday), *Oncophanes minutus* (Wesmael).

**Doryctinae:**

*Dendrosoter middendorffi* (Ratzeburg), *Ecphylus silesiacus* (Ratzeburg), *Spathius rubidus* (Rossi).

**Exothecinae:**

*Clinocentrus exsertor* (Nees), *Colastes aciculatus* Tobias*, *Colastes braconius* Haliday, *Colastes flavitarsis* (Thomson)*, *Colastes laevis* (Thomson)*, *Colastes pilosus* Belokobylskij*, *Hormius moniliatus* (Nees), *Hormius similis* Szépligeti, *Phaenodus pallipes* (Foerster), *Rhysipolis meditator* (Haliday).

**Braconinae:**

*Bracon abbreviator* Nees, *Bracon atrator* Nees*, *Bracon cingulator* Szépligeti*, *Bracon epitriptus* Marshall*, *Bracon erraticus* Wesmael*, *Bracon exhilarator* Nees*, *Bracon hebetor* Say, *Bracon hylobii* Ratzeburg, *Bracon intercessor* Nees*, *Bracon larvicida* Wesmael*, *Bracon leptus* Marshall*, *Bracon longicollis* Wesmael*, *Bracon minutator* (Fabricius), *Bracon osculator* Nees, *Bracon picticornis* Wesmael*, *Bracon praetermissus* Marshall*, *Bracon stabilis* Wesmael*, *Bracon terebella* Wesmael, *Bracon trucidator* Marshall*, *Bracon variator* Nees*, *Bracon variegator* Spinola*, *Coeloides abdominalis* (Zetterstedt), *Coeloides bostrichorum* Giraud.

**Rogadinae:**

*Aleiodes bicolor* (Spinola), *Aleiodes circumscriptus* (Nees), *Aleiodes coxalis* (Spinola), *Aleiodes ductor* (Thunberg), *Aleiodes esenbeckii* (Hartig), *Aleiodes fortipes* (Reinhard)*, *Aleiodes gasterator* (Jurine), *Aleiodes gastritor* (Thunberg)*, *Aleiodes nigricornis* Wesmael, *Aleiodes signatus* (Nees), *Aleiodes similis* (Curtis)*, *Aleiodes unipunctator* (Thunberg), *Aleiodes ungularis* (Thomson)*, *Aleiodes abraxanae* sp.n. van Achterberg*** (paratypes), *Aleiodes juniperi* sp.n. van Achterberg*** (paratypes).

**Gnamptodontinae:**

*Gnamptodon decoris* (Foerster)*, *Gnamptodon pumilio* (Nees).

**Opiinae:**

*Apodesmia circulator* (Nees)*, *Apodesmia similis* (Szépligeti)*, *Biosteres brevisulcus* (Thomson)*, *Demiostoma parvulum* (Wesmael)*, *Opius altimontanus* Fischer*, *Opius ambiguus* Wesmael*, *Opius attributus* Fischer*, *Opius caricivorae* Fischer*, *Opius crassipes* Wesmael*, *Opius levis* Wesmael*, *Opius lugens* Haliday*, *Opius pallipes* Wesmael, *Opius piceus* Thomson*, *Opius pygmaeator* (Nees)*, *Opius singularis* Wesmael, *Opius staryi* Fischer, *Phaedrotoma aethiops* (Haliday), *Phaedrotoma crassicrus* (Thomson), *Phaedrotoma curvata* (Fischer)*, *Phaedrotoma depeculator* Foerster, *Phaedrotoma exigua* (Wesmael)*, *Phaedrotoma rex* (Fischer), *Utetes caudatus* (Wesmael)*, *Utetes zelotes* (Marshall)*, *Xynobius comatus* (Wesmael)*.

**Alysiinae, Alysiini:**

*Alloea lonchopterae* Fischer*, *Alysia fuscipennis* Haliday*, *Alysia lucicola* Haliday, *Alysia luciella* Stelfox*, *Alysia similis* (Nees)*, *Alysia tipulae* (Scopoli)*, *Alysia truncator* (Nees)*, *Alysia umbrata* Stelfox, *Anisocyrta perdita* (Haliday)*, *Aphaereta elegans* Tobias, *Aphaereta falcigera* Graham*, *Aphaereta major* (Thomson)*, *Aphaereta scaptomyzae* Fischer, *Asobara tabida* (Nees)*, *Aspilota eurugosa* Fischer*, *Aspilota extremicornis* Fischer*, *Aspilota procreata* Fischer*, *Aspilota tetragona* Fischer*, *Aspilota stenogaster* Stelfox & Graham*, *Aspilota vernalis* Stelfox & Graham*, *Cratospila circe* (Haliday)*, *Dinotrema betae* (Bengtsson)*, *Dinotrema brevicauda* (Tobias)*, *Dinotrema carinatum* (Tobias)*, *Dinotrema contracticorne* (Fischer)*, *Dinotrema dimidiatum* (Thomson)*, *Dinotrema discoideum* (Fischer)*, *Dinotrema lineolum* (Thomson)*, *Dinotrema mesocaudatum* van Achterberg*, *Dinotrema oleraceum* (Tobias)*, *Dinotrema propodeale* (Tobias)*, *Dinotrema speculum* (Haliday)*, *Dinotrema spitzzickense* (Fischer)*, *Dinotrema tuberculatum* van Achterberg*, *Dinotrema varipes* (Tobias)*, *Dinotrema venustum* (Tobias)*, *Idiasta dichrocera* Königsmann*, *Idiasta subannellata* (Thomson)*, *Orthostigma longicorne* Königsmann, *Orthostigma longicubitale* Königsmann, *Orthostigma maculipes* (Haliday)*, *Orthostigma pumila* (Nees), *Orthostigma sculpturatum* Tobias*, *Orthostigma sordipes* Thomson, *Pentapleura angustula* (Haliday)*, *Pentapleura fuliginosa* (Haliday)*, *Pentapleura pumilio* (Nees)*, *Phaenocarpa angustiptera* Papp*, *Phaenocarpa conspurcator* (Haliday)*, *Phaenocarpa flavipes* (Haliday)*, *Phaenocarpa impugnata* Papp*, *Phaenocarpa livida* (Haliday)*, *Phaenocarpa ruflceps* (Nees)*, *Phaenocarpa styriaca* Fischer*, *Synaldis concolor* (Nees)*, *Synaldis distracta* (Nees)*, *Synaldis lacessiva* Fischer*, *Synaldis mandibulata* Fischer*, *Synaldis ultima* Fischer*, *Tanycarpa bicolor* (Nees), *Tanycarpa mitis* (Stelfox)*.

**Alysiinae, Dacnusini:**

*Agonia adducta* (Haliday)*, *Antrusa flavicoxa* (Thomson)*, *Antrusa melanocera* (Thomson), *Chorebus affinis* (Nees), *Chorebus anasella* (Stelfox)*, *Chorebus ares* (Nixon)*, *Chorebus asperrimus* Griffiths*, *Chorebus avesta* (Nixon)*, *Chorebus bathyzonus* (Marshall)*, *Chorebus brevicornis* (Thomson)*, *Chorebus daimenes* (Nixon)*, *Chorebus dirona* (Nixon)*, *Chorebus fordi* (Nixon)*, *Chorebus freya* (Nixon)*, *Chorebus ganesa* (Nixon)*, *Chorebus gnaphalii* Griffiths*, *Chorebus lateralis* (Haliday), *Chorebus misellus* (Marshall)*, *Chorebus nobilis* Griffiths*, *Chorebus nydia* (Nixon)*, *Chorebus ovalis* (Marshall)*, *Chorebus polygoni* Griffiths*, *Chorebus posticus* (Haliday)*, *Chorebus pseudomisellus* Griffiths*, *Chorebus senilis* (Nees)*, *Chorebus siniffa* (Nixon)*, *Chorebus subfuscus* Griffiths*, *Chorebus tanis* (Nixon)*, *Chorebus tenellae* Griffiths*, *Chorebus thusa* (Nixon)*, *Chorebus xanthospidae* Griffiths*, *Coelinidea elegans* (Curtis)*, *Coelinidea nigra* (Nees)*, *Coelinius parvulus* (Nees)*, *Coloneura arestor* (Nixon)*, *Coloneura ate* (Nixon)**, *Coloneura danica* Griffiths**, *Dacnusa areolaris* (Nees)*, *Dacnusa austriaca* (Fischer)*, *Dacnusa confinis* Ruthe*, *Dacnusa faeroeensis* (Roman), *Dacnusa laevipectus* Thomson, *Dacnusa liopleuris* Thomson*, *Dacnusa longithorax* (Tobias)*, *Dacnusa maculipes* Thomson*, *Dacnusa marica* (Nixon)*, *Dacnusa pubescens* (Curtis), *Dacnusa tarsalis* Thomson*, *Exotela cyclogaster* Foerster, *Exotela hera* (Nixon), *Exotela sonchina* Griffiths, *Exotela umbellina* (Nixon), *Saropspopovi* Tobias*, *Protodacnusa tristis* (Nees)*.

**Helconinae:**

*Diospilus dilatatus* Thomson, *Diospilus oleraceus* Haliday, *Helconidea dentator* (Fabricius), *Lestricus secalis* (Linnaeus).

**Brachistinae:**

*Eubazus longicaudis* (Ratzeburg), *Eubazus pallipes* Nees*, *Eubazus parvulus* (Ruthe)*, *Eubazus semirugosus* (Nees)*, *Triaspis floricola* (Wesmael)*, *Triaspis lugubris* Šnoflák, *Triaspis obscurella* (Nees)*, *Triaspis pallipes* (Nees)*.

**Euphorinae:**

*Blacus exilis* (Nees), *Blacus humilis* (Nees), *Blacus instabilis* Ruthe, *Blacus longipennis* (Gravenhorst)*, *Blacus leptostigma* Ruthe*, *Blacus maculipes* Wesmael, *Blacus nigricornis* Haeselbarth, *Blacus radialis* Haeselbarth, *Blacus ruficornis* (Nees), *Centistes collaris* (Thomson)*, *Centistes edentatus* (Haliday), *Dinocampus coccinellae* (Schrank), *Euphorus pallidistigma* Curtis*, *Leiophron clypealis* Tobias*, *Meteorus cinctellus* (Spinola)*, *Meteorus consimilis* (Nees)*, *Meteorus heliophilus* Fischer*, *Meteorus ictericus* (Nees), *Meteorus micropterus* (Haliday)*, *Meteorus obsoletus* (Wesmael), *Meteorus pulchricornis* (Wesmael), *Meteorus rubens* (Nees), *Meteorus versicolor* (Wesmael), *Meteorus unicolor* (Wesmael), *Myiocephalus boops* (Wesmael)*, *Myiocephalus niger* Fischer*, *Perilitus brevicollis* Haliday*, *Perilitus rutilus* (Nees), *Peristenus nitidus* (Curtis)*, *Peristenus pallipes* (Curtis)*, *Peristenus picipes* (Curtis)*, *Pygostolus falcatus* (Nees)*, *Spathicopis flavocephala* van Achterberg*, *Streblocera flaviceps* (Marshall)*, *Syntretus conterminus* (Nees)*, *Townesilitus bicolor* (Wesmael), *Townesilitus deceptor* (Wesmael)*, *Zele albiditarsus* Curtis, *Zele deceptor* (Wesmael).

**Agathidinae:**

*Agathis breviseta* Nees*, *Agathis tibialis* Nees*, *Agathis montana* Shestakov*, *Bassus cingulipes* (Nees)*, *Bassus conspicuus* (Wesmael)*, *Bassus pumilus* (Ratzeburg)*, *Earinus gloriatorius* (Panzer)*.

**Orgilinae:**

*Orgilus laevigatus* (Nees)*, *Orgilus obscurator* (Nees), *Orgilus pimpinellae* Niezabitowski.

**Macrocentrinae:**

*Macrocentrus bicolor* Curtis, *Macrocentrus collaris* (Spinola), *Macrocentrus flavus* Snellen van Vollenhoven*, *Macrocentrus pallipes* (Nees), *Macrocentrus resinellae* (Linnaeus).

**Homolobinae:**

*Homolobus discolor* (Wesmael), *Homolobus infumator* (Lyle).

**Charmontinae:**

*Charmon extensor* (Linnaeus).

**Adeliinae:**

*Adelius subfasciatus* Haliday.

**Miracinae:**

*Mirax rufilabris* Haliday.

**Cheloninae:**

*Ascogaster abdominator* (Dahlbom), *Ascogaster bidentula* Wesmael, *Ascogaster klugii* (Nees) *Ascogaster rufipes* (Latreille), *Ascogaster similis* (Nees)*, *Chelonus asiaticus* Telenga*, *Chelonus scabrator* (Fabricius), *Microchelonus basalis* (Curtis), *Microchelonus contractus* (Nees), *Microchelonus elachistae* Tobias, *Microchelonus gravenhorstii* (Nees), *Microchelonus* *karadagi* Tobias, *Microchelonus koponeni* Tobias, *Microchelonus microphthalmus* (Wesmael)*, *Microchelonus pedator* (Dahlbom), *Microchelonus pusillus* Szépligeti, *Microchelonus subpedator* Tobias, *Microchelonus temporalis* Tobias, *Microchelonus vickae* Lozan et Tobias, *Phanerotoma bilinea* Lyle.

**Microgastrinae:**

*Apanteles arsiba* Nixon*, *Apanteles ater* (Ratzeburg)*, *Apanteles atreus* Nixon*, *Apanteles brunnistigma* Abdinbekova*, *Apanteles corvinus* Reinhard*, *Apanteles decorus* (Haliday), *Apanteles imperator* Wilkinson, *Apanteles infimus* (Haliday), *Apanteles laevigatoides* Nixon, *Apanteles lenea* Nixon*, *Apanteles longicalcar* Thomson*, *Apanteles mycale* Nixon*, *Apanteles punctiger* (Wesmael)*, *Apanteles sicarius* Marshall*, *Apanteles tedellae* Nixon, *Apanteles viminetorum* (Wesmael), *Apanteles xanthostigma* (Haliday), *Cotesia acuminata* (Reinhard)*, *Cotesia analis* (Nees)*, *Cotesia euryale* (Nixon)*, *Cotesia gastropachae* (Bouché), *Cotesia hyphantriae* (Riley)*, *Cotesia lineola* (Curtis)*, *Cotesia melanoscela* (Ratzeburg), *Cotesia ordinaria* (Ratzeburg)*, *Cotesia praepotens* (Haliday)*, *Cotesia tetrica* (Reinhard), *Cotesia tibialis* (Curtis), *Cotesia vanessae* (Reinhard)*, *Cotesia vestalis* (Haliday)*, *Cotesia zygaenarum* (Marshall), *Deuterixys carbonaria* (Wesmael)*, *Diolcogaster hinzi* (Nixon)*, *Diolcogaster minuta* (Reinhard), *Hygroplitis rugulosus* (Nees)*, *Microgaster acilia* Nixon*, *Microgaster alebion* Nixon*, *Microgaster meridiana* Haliday*, *Microgaster globata* (Linnaeus)*, *Microgaster hospes* Marshall, *Microgaster postica* Nees*, *Microgaster stictica* Ruthe*, *Microgaster subcompleta* Nees, *Microgaster messoria* Haliday*, *Microplitis deprimator* (Fabricius), *Microplitis fordi* Nixon*, *Microplitis fulvicornis* (Wesmael)*, *Microplitis mediator* (Haliday), *Microplitis spinolae* (Nees)*, *Microplitis stenuus* Reinhard*, *Microplitis tuberculifer* (Wesmael), *Microplitis viduus* (Ruthe)*, *Microplitis xanthopus* (Ruthe)*, *Protapanteles anchistades* (Nixon)*, *Protapanteles callidus* (Haliday)*, *Protapanteles compressiventris* (Muesebeck)*, *Protapanteles falcatus* (Nees)*, *Protapanteles fraternus* (Reinhard)*, *Protapanteles fulvipes* (Haliday), *Protapanteles liparidis* (Bouché), *Protapanteles pallipes* (Reinhard)*, *Protapanteles trinagulator* (Wesmael)*, *Protapanteles vitripennis* (Curtis)*.

* new faunistic record for Czech Republic

** new faunistic record for Central Europe

*** two new species of the genus *Aleiodes* will be described by the third author (C. van Achterberg) separately. Part of paratypes are from peatbogs.
